# Maternal Smoking during Pregnancy and DNA-Methylation in Children at Age 5.5 Years: Epigenome-Wide-Analysis in the European Childhood Obesity Project (CHOP)-Study

**DOI:** 10.1371/journal.pone.0155554

**Published:** 2016-05-12

**Authors:** Peter Rzehak, Richard Saffery, Eva Reischl, Marcela Covic, Simone Wahl, Veit Grote, Annick Xhonneux, Jean-Paul Langhendries, Natalia Ferre, Ricardo Closa-Monasterolo, Elvira Verduci, Enrica Riva, Piotr Socha, Dariusz Gruszfeld, Berthold Koletzko, European Childhood Obesity Trial Study group

**Affiliations:** 1 Division of Metabolic and Nutritional Medicine, Dr. von Hauner Children’s Hospital, Ludwig-Maximilians-University of Munich, University of Munich Medical Centre, Munich, Germany; 2 Cancer and Disease Epigenetics Research Group, Murdoch Childrens Research Institute, Royal Children's Hospital, Flemington Road, Parkville, 3052 Victoria Australia; 3 Research Unit of Molecular Epidemiology, Institute of Epidemiology II, Helmholtz Zentrum Muenchen, Munich, Germany; 4 CHC St Vincent, Liège-Rocourt, Belgium; 5 Universitat Rovira I Virgili, Reus, Spain; 6 University of Milano, Milano, Italy; 7 Children’s Memorial Health Institute, Warsaw, Poland; Emory University, UNITED STATES

## Abstract

Mounting evidence links prenatal exposure to maternal tobacco smoking with disruption of DNA methylation (DNAm) profile in the blood of infants. However, data on the postnatal stability of such DNAm signatures in childhood, as assessed by Epigenome Wide Association Studies (EWAS), are scarce. Objectives of this study were to investigate DNAm signatures associated with *in utero* tobacco smoke exposure beyond the 12^th^ week of gestation in whole blood of children at age 5.5 years, to replicate previous findings in young European and American children and to assess their biological role by exploring databases and enrichment analysis. DNA methylation was measured in blood of 366 children of the multicentre European Childhood Obesity Project Study using the Illumina Infinium HM450 Beadchip (HM450K). An EWAS was conducted using linear regression of methylation values at each CpG site against *in utero* smoke exposure, adjusted for study characteristics, biological and technical effects. Methylation levels at five HM450K probes in *MYO1G* (cg12803068, cg22132788, cg19089201), *CNTNAP2* (cg25949550), and *FRMD4A* (cg11813497) showed differential methylation that reached epigenome-wide significance according to the false-discovery-rate (FDR) criteria (*q-value<0*.*05*). Whereas cg25949550 showed decreased methylation (-2% DNAm ß-value), increased methylation was observed for the other probes (9%: cg12803068; 5%: cg22132788; 4%: cg19089201 and 4%: cg11813497) in exposed relative to non-exposed subjects. This study thus replicates previous findings in children ages 3 to 5, 7 and 17 and confirms the postnatal stability of *MYO1G*, *CNTNAP2* and *FRMD4A* differential methylation. The role of this differential methylation in mediating childhood phenotypes, previously associated with maternal smoking, requires further investigation.

## Introduction

There is growing evidence from both candidate gene [1] and Epigenome Wide Association Studies (EWAS) that prenatal exposure to maternal tobacco smoking substantially alters DNA methylation (DNAm) in newborn blood [2–6]. Evidence also exists for dosage, tissue-specific and timing of exposure effects. Importantly, several of these studies have reported similar findings, with changes in methylation consistently observed at *AHHR*, *CNTNAP2*, *CYP1A1*, *GFI1* and *MYO1G* at birth.

Recently, the postnatal stability of maternal smoking-associated differential methylation has been investigated. An EWAS using the Illumina Infinium HumanMethylation450 BeadChip (HM450K), conducted in 12- to 18-year old offspring of mothers who smoked during pregnancy [6] found persistence of differentially methylated regions (DMRs) in *MYO1G* and *CNTNAP2*. A similar study using longitudinal blood samples collected at birth, at age 7 and age 17 years [7], demonstrated that some smoking-associated DMRs are time-stable postnatally (*AHRR*, *MYO1G*, *CYP1A1*, *CNTNAP2)*, whereas others are not (*ATP9A*, *GFI1*, *KLF13*). Importantly, both the duration and intensity (cigarettes/day) appear important in the observed effect. Despite these findings, no equivalent EWAS has yet been carried out in blood of children younger than 7, except a very recent study in 3–5 year old US children [8] using EWAS data to replicate 26 differently methylated CpG sites previously reported in newborns [2].

In order to further explore the postnatal stability of maternal smoking induced epigenetic change in early childhood, we carried out an EWAS in 366 children at 5.5 years of age as part of the multicentre study of the European Childhood Obesity Project Study (CHOP) [9]. In addition, we explored biological databases and performed enrichment analysis to get some biological insight into the role of MYO1G, CNTNAP2 and FRMD4A.

## Results

Overall 5 CpG sites in genes *MYO1G* (three sites), *CNTNAP2* and *FRMD4A* showed epigenome-wide significance according to the FDR criteria (**Table 1**).

**Table 1:** Top 25 EWAS associations of differentially methylated CpG sites in children at age 5.5 years exposed to maternal smoking beyond week 12 of gestation.

CpG ID	CHR	Gene	Position (bp)	DNAm ß-value difference (SE)	*P*-value	FDR
cg12803068	7	*MYO1G*	45002919	0.09 (0.02)	7.15E-10	3.09E-04
cg25949550	7	*CNTNAP2*	145814306	-0.02 (0.00)	1.57E-09	3.38E-04
cg22132788	7	*MYO1G*	45002486	0.05 (0.01)	1.73E-08	2.49E-03
cg11813497	10	*FRMD4A*	14372879	0.04 (0.01)	2.17E-07	2.34E-02
cg19089201	7	*MYO1G*	45002287	0.04 (0.01)	5.69E-07	4.91E-02
cg27181554	16	*SEPX1*	1992734	-0.02 (0.00)	1.49E-06	9.35E-02
cg21376136	2	*CACNB4*	152830572	0.05 (0.01)	1.52E-06	9.35E-02
cg23222488	5		176755393	0.05 (0.01)	9.02E-06	4.77E-01
cg03480605	7	*NUB1*	151038446	0.04 (0.01)	9.95E-06	4.77E-01
cg17745339	5		176755199	0.05 (0.01)	1.21E-05	5.20E-01
cg00173010	7	*DAGLB*	6487342	0.00 (0.00)	1.81E-05	7.11E-01
cg10427827	12		132929654	0.01 (0.00)	2.54E-05	8.68E-01
cg25959131	3	*DOCK3*	50855436	0.04 (0.01)	2.95E-05	8.68E-01
cg15507334	10	*FRMD4A*	14372913	0.04 (0.01)	3.07E-05	8.68E-01
cg11025974	2	*CACNB4*	152830521	0.03 (0.01)	3.17E-05	8.68E-01
cg26624744	5	*CYFIP2*	156692507	-0.02 (0.01)	3.34E-05	8.68E-01
cg03287111	2	*GLI2*	121625634	0.04 (0.01)	3.59E-05	8.68E-01
cg15775217	12	*TXNRD1*	104676774	0.18 (0.04)	3.74E-05	8.68E-01
cg04180046	7	*MYO1G*	45002736	0.05 (0.01)	3.82E-05	8.68E-01
cg25292698	18	*MYL12A*	3247809	-0.01 (0.00)	4.48E-05	9.66E-01
cg14362178	2		79007750	-0.07 (0.02)	4.90E-05	1.00E+00
cg05655457	11	*OR5B17*	58127944	-0.04 (0.01)	5.33E-05	1.00E+00
cg08237309	16	*PKD1*	2146423	0.00 (0.00)	5.53E-05	1.00E+00
cg21428148	18	*NDUFV2*	9102454	0.00 (0.00)	5.81E-05	1.00E+00
cg18882449	10	*NT5C2*	104885122	0.05 (0.01)	6.17E-05	1.00E+00

Table is based on regression analysis of 431313 CpGs in 366 children. *CpG ID*: CpG site identification number and *CHR*: chromosome according to annotation. Gene: CpG related gene name according to annotation; *Position (bp)*: Chromosome position is based on NCBI Human Reference Genome Assembly Build 37. DNAm ß-value difference is the adjusted mean difference in differentially methylated ß-values among *in utero* tobacco exposed vs. not exposed children for those 25 CpG sites identified by the M-value based EWAS regression models described below; e.g. 0.09 means that the difference in the proportion of DNA methylation between exposed and unexposed children is 09. *P*-value: uncorrected *p*-value obtained by standard linear regression of M-values of DNA methylation at respective CpG site (outcome) on indicator coded *in utero* tobacco exposure by maternal smoking during pregnancy (main predictor) adjusted for sex, age at blood draw, indicator coded country of study centre, maternal education, exposure to parental smoking at age 4 years, the estimated proportions of six major white blood cell (WBC) types, CD4+ T cells, CD8+ T cells, B cells, NK cells, monocytes and granulocytes, and the 30 principal components (PC) derived from control probes (see methods section). *FDR*: *p*-value corrected for multiple testing according to Benjamin-Hochberg (false discovery rate).

**Fig 1:**
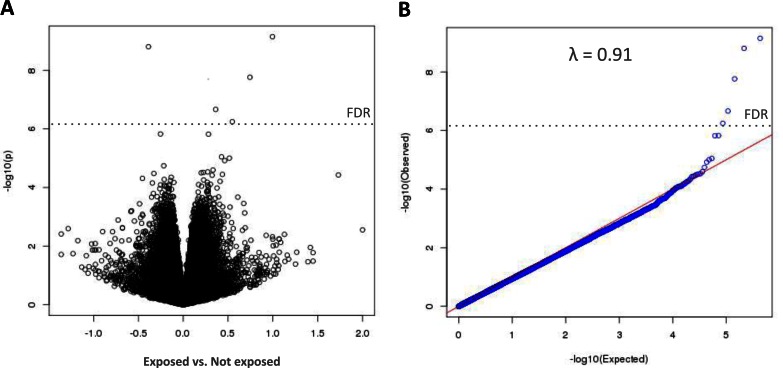
**Volcano plot (A) and QQ-plot (B) of 433 313 differentially methylated CpG sites for 366 children at age 5.5 exposed vs. not-exposed to maternal smoking beyond week 12 of gestation.** Figures are based on linear regression of M-values on exposure variable and adjustment factors.

EWAS significance and effect size of 431 313 analysed CpG sites are illustrated also by volcano plot and QQ-plot **(Fig 1)**.

**Fig 2:**
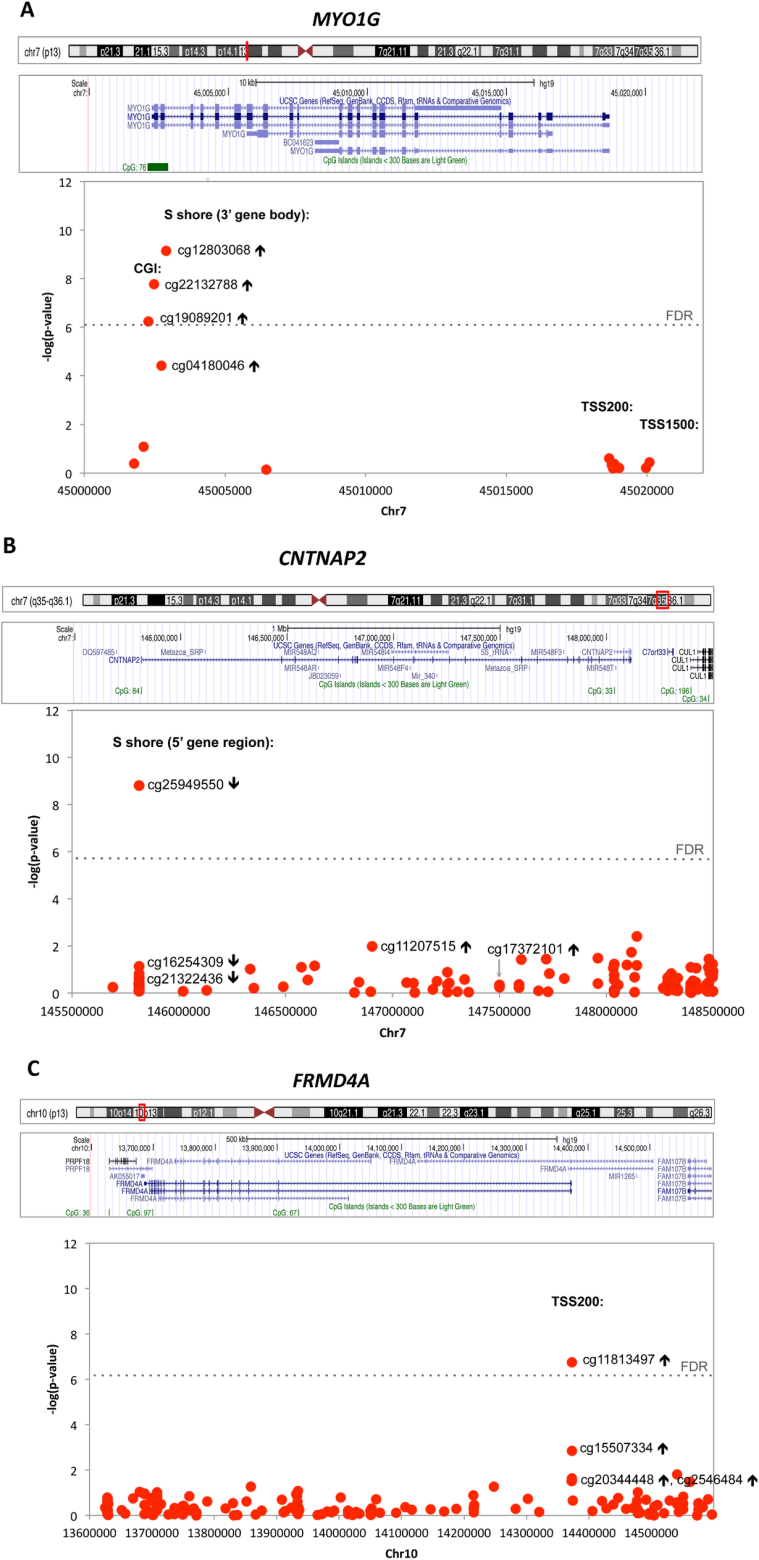
**Regional association of CpG methylation at *MYO1G* (A), *CNTNAP2* (B) and *FRMD4A* (C) loci with prenatal smoking status.** The direction of differential methylation is marked with arrows (↑ increase or ↓ decrease). Regional association plots are shown using the gene map (UCSC genome browser, hg19) with a graph of–log_10_ *p*-values on the y-axis, the nucleotide position on the x-axis, and the position of selected Illumina Infinium HumanMethylation 450 BeadChip probes. Abbreviations: CGI: CpG island; TSS: transcription start site; FDR: false discovery rate *p*-value.

The location of differentially methylated CpG sites in *MYO1G*, *CNTNAP2* and *FRMD4A* is depicted in **Fig 2**.

The three significant differentially methylated CpG sites in *MYO1G* were situated at the 3’ gene region, in a CpG island (cg22132788, cg19089201) and the 2kb region downstream of this CpG island, i.e. in an S shore (cg12803068) (**Fig 2A**). The significantly methylated CpG in *CNTNAP2* gene was situated in S shore (cg25949550), close to the transcription start site (TSS) (**Fig 2B**). One significantly methylated CpG site in *FRMD4A* (cg11813497) was located downstream of the TSS (**Fig 2C**). Amongst the top 25 differentially methylated CpG sites in association with maternal smoking were other previously reported CpGs in *MYO1G* (cg04180046) and *FRMD4A* (cg15507334) genes (**Table 1**), but these did not survive correction for multiple testing.

CpGs showing significant differential methylation in association with maternal smoking after week 12 of gestation generally showed positive associations; *MYO1G*, +9% at cg12803068, +5% at cg22132788 and +4% at cg19089201; FRMD4A, +4% at cg11813497. In addition, cg04180046 at the 3’region of *MYO1G* showed a +5% difference and cg15507334 at the 5’ region of *FRMD4A* a +4% difference in mean CpG methylation between exposed and unexposed children but both did not reach FDR significance.

In contrast, cg25949550 in the S shore of *CNTNAP2* gene showed a difference of minus 2% in mean DNA methylation between exposed and unexposed children (**Table 1**).

Direction and effect sizes of differential methylations in *MYO1G* and *CNTNAP2* genes found in the CHOP cohort (5.5 years) were quite similar with those from other EWAS based on Illumina 450K arrays from SEED (5 years), ALSPAC (7.5 and 17.7 years) and SYS (15.6 years) cohorts (**S1 Table**) [6–8].

### Functional Network and Enrichment Analysis

The PANTHER Classification System (http://pantherdb.org/index.jsp) [10] showed in the predicted network of 100 proteins associated with MYO1G, CNTNAP2 and FRMD4A (**S2 Table**) created by GeneMANIA ((http://genemania.org/) [11] a more than 5-fold enrichment for the following pathways: Nicotine pharmacodynamics pathway (P06587), Dopamine receptor mediated signalling pathway (P05912), Axon-guidance mediated by semaphorins (P00007) and Ras pathway (P04393) (**S3 Table**).

Some of the strongest overrepresented GO terms were protein localization to juxtaparanode region of axon (GO:0071205), neuron recognition (GO:0008038), axonogenesis (GO:0050770), cell-substrate adhesion (GO:0031589), neuron projection guidance (GO:0097485) and included other biological processes linked to cytoskeleton organization, cell migration and motility (**S4 Table**). For a complete list of significant GO terms linked to molecular function and cellular localisation see **S5**and **S6 Tables**.

PANTHER protein classes in this network were >5-fold enriched (Bonferroni-adjusted *P*-value<0.05) for actin binding motor proteins (PC00040), cell junction proteins (PC00070), actin family cytoskeletal proteins (PC00041) and G-protein modulators (PC00022) see **S7 Table**.

## Discussion

This study aimed to identify associations between *in utero* exposure to maternal smoking and DNA methylation levels in blood of 366 European children of age 5.5 years. Overall five CpG sites in gene regions *MYO1G* (cg12803068, cg22132788, cg19089201), *CNTNAP2* (cg25949550) and *FRMD4A* (cg11813497) showed epigenome-wide significance. Interestingly, the CpG sites we identified in the *CNTNAP2* and *MYO1G* genes were the same as those previously reported in newborns [2], 3–5 year old children [8], for adolescents at age 12–16 years [6] and longitudinally in newborns, 7 year old children and 17 year old adolescents [7]. Moreover, the effect size and direction of effect were more or less equivalent across these studies, highlighting the reproducibility of findings and postnatal stability of *in utero* smoking exposure–associated DNA methylation disruption in these genes.

The differentially methylated CpG site cg11813497 in the promoter region of *FRMD4A* in our study has previously been reported in a single EWAS in blood of newborns with the largest proportion of smoking mothers yet examined [3]. However, whereas that study identified 3 other *FRMD4A* related CpG sites (cg2034448, cg25464840, cg15507334) these were not significantly differentially methylated in our dataset [3]. One of these sites (cg25464840) was also reported in an EWAS of 5–12 year old children [12]. As only one of these previous reported CpG sites in *FRMD4A* (cg15507334) was among the top 25 ranked *p*-values in our study, it is interesting to speculate that whereas differential methylation at CpG site cg11813497 may show throughout childhood, this may not be the case for the other 2 CpG sites in this gene. However, equally likely is that the reduced power of our study, relative to previous studies [3] played a role in our findings.

Several other differently methylated regions (DMR) in children or adolescents associated with maternal smoking during pregnancy in previous EWAS, e.g. CpGs related to *AHRR* or *CYP1A1* in the study [6,7] did not show significance in our EWAS analyses.

### Biological meaning of CpG sites sensitive to maternal smoking during pregnancy

Prenatal tobacco smoke exposure is associated with a wide range of adverse health outcomes in offspring. Children of smoking mothers have an increased risk of speech-processing and attention control deficits [13,14], autism [15], allergy [16], asthma [17], overweight and obesity [18] and nicotine dependency [19]. Interestingly, MYO1G, CNTNAP2 and FRMD4A gene products have been previously been associated with many of these outcomes and differential DNA methylation across their gene loci has been consistently replicated in the blood of newborns or youth exposed to *in utero* tobacco smoke. However, the functional relevance of these DMRs at *CNTNAP2*, *MYO1G* and *FRMD4* is limited. Therefore, we investigated publicly available epigenome and transcriptome databases and performed functional network and enrichment analysis to gain insights into the potential functional relevance of the observed differential methylation in these genes.

#### MYO1G

Smoking-associated increased methylation at *MYO1G* (cg19089201, cg22132788, cg04180046, and cg12803068) is located in a CpG island and S shore in the 3’ gene region (**S1 Fig**). It has been detected both in offspring exposed to prenatal smoking [2,5–8] and in adult smokers [20–24].

Increased methylation in this gene region might correlate with increased *MYO1G* transcription according to three lines of evidence. First, the DNA methylation and gene expression database MethHC [25] shows that in urogenital cancer the methylation at 3’ gene region of *MYO1G* positively correlates with transcription, contrary to the promoter/5’UTR region methylation (**S2 Fig**). Second, The Encyclopedia of DNA Elements (ENCODE) [26] evidence of DNA methylation in three blood and endothelial cell lines (GM12878, K562 and HUVEC) by Illumina 450K shows that high methylation (>60%) in the 3’ *MYO1G* region and low methylation (<20%) in the 5’ gene region correlate with high *MYO1G* transcription in GM12878 (**S1 Fig**) [27]. On the contrary, in K562 and HUVEC cell lines where *MYO1G* transcription is absent [28], methylation at the 3’ gene region is low (<20% in HUVEC) while the 5’ gene region methylation is high (>60% in K562 and HUVEC) (**S1 Fig**). Third, methylation in the 3’ *MYO1G* gene body (cg04180046) was recently reported to positively correlate with *MYO1G* transcription in leukocytes of 144 healthy Chinese adults in an EWAS of smoking [24]. Thus, these data support the observation that, unlike promoter methylation, gene body methylation in *MYO1G* might correlate with its active transcription [29].

The 3’ *MYO1G* gene region might have a regulatory role since it overlaps with monomethylated and trimethylated lysine 4 on histone H3 (H3K4) and binding sites for Myc, Max, YY and USF transcriptional factors (**S3 Fig**). It also binds transcriptional repressors such as EZH2, a histone-lysine N-methyltransferase involved in regulation of histone and DNA methylation [30] and CTCF, an insulator protein that affects mRNA splicing, promoter-enhancer looping and whose binding affinity is modulated by DNA methylation [31].

Exposure to nicotine may change leukocyte numbers and increase leukocyte-endothelial adhesion [32]. This goes in line with GO terms in our pathway analysis such as cell migration (GO:0016477), localization of cell (GO:0051674), cell motility (GO:0048870), movement of cell or subcellular component (GO:0006928) and cell adhesion (GO: 0007155). Moreover, MYO1G might be implicated in transmission of smoking effects on cardiovascular system [33] since this type I unconventional myosin is specifically expressed in the plasma membrane of B and T lymphocytes and mast cells [28,34] and regulates leukocyte adhesion, mobility and phagocytosis [35–37].

Sensory perception of sound (GO: 0007605) was another overrepresented biological term in our pathway analysis, which might by associated with impaired auditory processing in infants exposed to prenatal smoking [38]. However, this overrepresentation might be also due to the presence of other nonconventional myosins such as MYO1B, MYO1C and MYO1E in our interaction network. Unlike MYO1G, they are also expressed in sensory cells of the inner ear [39] and their mutations have been associated with hearing loss [40].

Based on current knowledge, it would be of interest to understand the mechanisms that keep 3’ MYO1G gene region differentially methylated even after the withdrawal of tobacco toxins and what role this plays for phenotypes related to child development.

#### CNTNAP2

Decreased methylation at *CNTNAP2* (cg25949550) has been reported both in offspring exposed to prenatal smoking [2,5–7] and in adults [22,24]. It is located in S shore, ~800 bp downstream of the transcriptional start site, and overlaps with binding sites of several transcriptional repressors such as SIN3A, CTCF, REST and CTBP2 (**S4 Fig)**. Adult smokers also show decreased methylation at nearby cg21322436 [22,24] and cg16254309 [41]. These two CpGs are located in N shores and S shores (0-2kb regions upstream and downstream of CGI, respectively) in the 5’ gene region (**Fig 2B, S4 Fig**). In addition, adult smokers also show increased methylation at cg11207515 and cg1737210 that are located >1 Mb (mega base pairs) downstream of the *CNTNAP2* transcriptional start site (**Fig 2B**) [22–24]. These DMRs found in blood of adult smokers have not yet been found in offspring in association with the exposure to prenatal tobacco smoke.

Given the evidence from Gene Expression Atlas [42] that tobacco down-regulates *CNTNAP2* transcription in cancerous human lung tissue (E-GEOD-13309) and normal human bronchial epithelial cells (E-GEOD-10718) [43,44], we speculate that *CNTNAP2* transcription could also be downregulated in individuals exposed to tobacco smoke. Whether this could be mediated through methylation decrease in one CpG and increased recruitment of transcriptional repressors remains to be addressed. This might even be detectable in blood since CNTNAP2 protein expression has also been found in B lymphocytes and endothelial cells [45,46].

CNTNAP2 is a cell adhesion protein whose decreased expression leads to aberrant neuronal migration and was included in some of our overrepresented GO terms such as ‘protein localization to juxtaparanode region of axon’ (GO:0071205), ‘neuron recognition’ (GO:0008038) and ‘neuron projection development’ (GO:0031175). Since CNTNAP2 mutations have been linked with impaired language development, autism spectrum disorder and intellectual disability [47], it is possible that the prenatal smoking-associated methylation in *CNTNAP2* and its persistency until at least adolescence [6,7] might represent an additional mechanism contributing to impaired neurodevelopment in children exposed to *in utero* tobacco smoke.

#### FRMD4A

Increased methylation in *FRMD4A* (cg20344448, cg11813497, cg25464840 and cg15507334) has been associated with prenatal smoking in newborns [3,5] and children [12] but not observed in adult smokers. These CpGs are situated in the potential promoter/5’UTR region (**Fig 2C)**, which in neuroblastoma cells binds GATA-2 (**S5 Fig**), a hematopoietic transcription factor that regulates proliferation of blood cell lineages. Interestingly, binding of GATA-2 to target genes is prevented by DNA methylation during lymphoid differentiation [48]. *In vitro* experiments suggest that smoking might modulate *FRMD4A* transcription since *FRMD4A* expression in human bronchial cells treated with tobacco smoke condensate was upregulated (E-GEOD-14383) [49]. However, no relation between DNA methylation and expression of *FRMD4A* was found in 526 adults used for replication of EWAS findings related to prenatal smoking exposure in older children [12].

Prenatal exposure to nicotine exerts strong effects on cytoskeleton reorganization and increases expression of cytoskeletal proteins [50]. Our functional network analysis was overrepresented for 2 GO terms related to morphogenesis (GO:0009653 and GO:0032989), both of which included FRMD4A and MYO1G. FRMD4A is a scaffold protein that regulates epithelial polarization and cell-cell junctions [51] and its upregulation increases intercellular adhesion [52]. The influence of prenatal nicotine on cytoskeleton organization is further supported by overrepresented PANTHER Pathways such as Dopamine receptor mediated signalling (P05912) and Nicotine pharmacodynamics (P06587) pathways. Although they did not include FRMD4A, the EPB41L1 erythrocyte cytoskeleton protein that shares the FERM-C protein domain with FRMD4A was found in both of these pathways. Interestingly, EPB41L1 and its related protein EPB41L3 interact with CNTNAP2 in brain where they may anchor this axonal transmembrane protein to the actin cytoskeleton [53].

The relevance of promoter DNA methylation on FRMD4A function in development remains unclear. Since mutations in *FRMD4A* gene locus associate with nicotine dependence [54], prenatal smoking-induced DNA methylation of *FRMD4A* that persists until childhood might be an additional mechanism contributing to increased nicotine dependency of offspring exposed to in utero tobacco smoke [19,55].

### Strengths and limitations

With the exception of a very recent replication study in 3–5 year old US children [8] our study is the first comprehensive EWAS with the HM450K BeadChip addressing the effect of continued in utero tobacco smoke exposure in children of age 5.5 years from four different European populations (Belgian, German, Italian and Spanish).

This complements previous EWAS that were conducted in British, Norwegian and French-Canadian populations [6,7,12] at different ages from birth to age 17 years. Although our sample size is reasonable large (n = 366), as is the exposure group (n = 58), it is likely that it was somewhat underpowered to detect smaller effects sizes at all genomic loci likely to be sensitive to methylation disruption in association with maternal smoking *in utero*. Future meta-analyses across multiple cohorts using the HM450K platform should now be performed to further define the scope of methylation disruption associated with this exposure in pregnancy.

## Conclusions

This EWAS showed effects of continued *in utero* tobacco smoke exposure beyond 12^th^ week of gestation on the methylation profile of children at age 5.5 years in Belgian, German, Italian and Spanish populations. We replicated previous findings confirming the postnatal stability of methylation disruption at *MYO1G* and *CNTNAP2* genes in association with *in utero* exposure to maternal smoking throughout pregnancy and confirmed differential methylation of a CpG site in *FRMD4A* that was previously found only in newborns.

The consistent replication of these methylated CpG sites and the exploration of biological databases combined with enrichment analysis suggests that their biological function and health-related phenotypes should be further explored.

## Material and Methods

### Study design and analysed population

The study population used is a subset of 366 children out of 543 children aged 5.5 years from study centres in Germany, Belgium, Italy and Spain of the European Childhood Obesity Trial Study (CHOP) registered at clinicaltrials.gov as NCT00338689 and URL: http://clinicaltrials.gov/ct2/show/NCT00338689?term=NCT00338689&rank=1.

Inclusion criteria were availability of blood buffy coats collected at age 5.5, valid exposure data on maternal smoking during pregnancy and information on basic characteristics of mother and offspring (sex, age of blood draw, country of study centre).

Characteristics of the analysed study population are listed in **Table 2**.

**Table 2:** Characteristics of the analysed population.

Characteristic	not exposed	exposed	exposed vs. not	Total
	(n = 308)	(n = 58)	*p*-value	(n = 366)
Girls (n (%))	163 (52.9)	25 (43.1)	0.172	188 (51.4)
Child's age at blood draw (months)	66.4 ± 0.8	66.6 ± 0.8	0.177	66.4 ± 0.8
Girls birthweight (kg)	3.3 ± 0.3	3.0 ± 0.3	< .0001	3.2 ± 0.3
Boys birthweight (kg)	3.3 ± 0.3	3.2 ± 0.4	0.030	3.3 ± 0.3
Child formula vs. breastfed (%)	190 (61.7)	52 (89.7)	< .0002	242 (66.1)
Length of gestation (week)	39.8 ± 1.2	39.8 ± 1.4	0.920	39.8 ± 1.2
Maternal age at delivery (years)	31.9 ± 4.2	32.6 ± 4.2	0.276	32 ± 4.2
Maternal education				
Low (n (%))	38 (12.4)	24 (41.4)	< .0001	62 (17)
Middle (n (%))	163 (53.3)	27 (46.6)	0.349	190 (52.2)
High (n (%))	105 (34.3)	7 (12.1)	0.002	112 (30.8)
Country of study centre				
Germany (n (%))	29 (9.4)	2 (3.4)	0.152	31 (8.5)
Belgium (n (%))	58 (18.8)	10 (17.2)	0.775	68 (18.6)
Italy (n (%))	122 (39.6)	14 (24.1)	0.028	136 (37.2)
Spain (n (%))	99 (32.1)	32 (55.2)	0.001	131 (35.8)
Smoke exposure at age 4 years				
by child's mother (n (%))	31 (10.1)	40 (69.0)	< .0001	71 (19.4)
by child's father (n (%))	75 (24.4)	27 (46.6)	0.001	102 (27.9)

*Exposed*: Child exposed to any *in utero* tobacco smoking by maternal smoking during pregnancy beyond 12^th^ week of gestation; *Not Exposed*: no exposure to *in utero* tobacco smoking at any time during pregnancy. *P-value*: test of percent or mean difference in study population characteristic among exposed vs. not exposed group either by chi-square test or t-test.

Details on this ongoing prospective nutritional intervention trial with n = 1678 enrolled healthy infants around birth (n = 550 higher protein formula, n = 540 lower protein formula, n = 588 breastfed) have been published previously [9,56].

### Ethics Statement

The study was conducted according to the principles expressed in the Declaration of Helsinki. The local ethics committees of each study center approved all study procedures: Belgium (Comitè d’Ethique de L’Hopital Universitaire des Enfants Reine Fabiola; no. CEH 14/02), Germany (Bayerische Landesärztekammer Ethik-Kommission; no. 02070), Italy (Azienda Ospedaliera San Paolo Comitato Etico; no. 14/2002), Poland (Instytut Pomnik–Centrum Zdrowia Dziecka Komitet Etyczny; no 243/KE/2001), and Spain (Comité ético de investigación clinica del Hospital Universitario de Tarragona Joan XXIII). Written informed parental consent was obtained for each infant.

### Assessment of child’s smoking exposure during pregnancy

*In utero* tobacco exposure was derived from maternal questionnaires administered during the first 8 weeks after delivery:

“Did the child's mother smoke **during early** pregnancy (up to the 12^th^ week of gestation)?”

“Did the child's mother smoke **in the further course of pregnancy** (beyond the 12^th^ week of gestation)?”

Only 18 of 76 mothers who reported smoking in pregnancy ceased after the 12^th^ week of gestation. Given previous findings demonstrating the necessity for prolonged exposure [8,57], results are reported only for mothers who smoked throughout pregnancy. The analysed exposure variable was coded 1, if the child was exposed to maternal smoking beyond the 12^th^ gestational week and 0 if not exposed beyond this point. Exposure was set to missing if a child was exposed only up to 12^th^ week of gestation.

### Measurement of epigenome-wide DNA methylation

During child’s physical examination at age 5.5 years blood was drawn to collect peripheral blood cells from buffy coats. Genomic DNA was extracted from buffy coats using a standard precipitation procedure. Bisulfite conversion of 800 ng genomic DNA was conducted with the EZ-96 DNA Methylation Kit (Zymo Research, Irvine, Ca; USA) and converted DNA samples were hybridised on the Infinium HumanMethylation450 BeadChip (HM450K) according to the manufactures instructions (Illumina Inc., San Diego, USA). DNA extraction, bisulfite conversion and methylation analysis were performed at the Genome Analysis Center of Helmholtz Zentrum Muenchen, Munich, Germany.

DNA samples were randomly assigned to each of the 33 HM450K slides each containing 12 arrays and processed in one batch by the same laboratory staff to reduce potential batch effects. The HM450K platform measures methylation at 485577 CpG sites located throughout the genome [58]. It covers 21231 (99%) genes of the UCSC RefGenes and has a coverage of 96% of the CpG islands (CGI), 92% of the CGI shores (0–2 kb from CGI), 86% of the CGI shelves (2–4 kb from CGI) and interrogates 16232 differentially methylated regions (DMR).

DNA methylation (DNAm) status at each HM450K probe was determined as methylation ß-value derived from the intensity ratio of the methylated allele (M) to the sum of intensities of the unmethylated allele (U) and the methylated allele (M) plus an offset of 100 [58]. As individual blood samples contain many genomic copies of each CpG site, the calculated ß-value can be interpreted as the percentage of methylation at that interrogated CpG site for that specific sample with a range from 0 (= completely unmethylated) to 1 (= completely methylated). Methylation ß-values were converted to M-values for statistical analyses by taking the base 2 logarithm of the ratio of the raw methylation ß-value and 1 minus the ß-value [59].

### Quality control and normalisation of methylation data

Data pre-processing and normalisation were performed mainly according to the approach of Touleimat and Tost [60] with some adaptions e.g. the beta-mixture quantile normalization (BMIQ) step of the ß-values [61] as recommend by a recent review of pre-processing and normalisation procedures [62].

Data were only retained for probes that had signals from at least 3 functional beads on the array and had detection *p*-values *p*≤0.01. Moreover, only samples with at least 80% significant probe methylation signals per sample were retained. Colour bias correction using smooth quantile normalisation, and background adjustment based on negative control probes were conducted separately for the two colour channels with R-package lumi. Lumi was also used for signal background correction by subtraction of the negative control probe signal. Several features of the process of pre-processing used in this study were described previously in more detail [63]. In contrast to previous approaches [60], no probe filtering according to proximity of CpG site with SNPs of minor allele frequency ≥5% within 50bp was conducted. In addition, probes on the X and Y chromosomes were not excluded. However, after BMIQ-normalisation and removal of duplicates, identified cross-binding probes were excluded as previously described [64] leaving a final data set of 431313 CpG methylation values in each of 366 children for EWAS analysis.

Batch effects and other technical noise was assessed by principal components analysis (PCA) of control-probes and accounted for by adjustment for principal components (PC) in the EWAS analysis [65]. Heterogeneity of cell mixture distribution in the samples were assessed and accounted for by Houseman’s method [66]. In detail, we used the validation data set consisting of purified cell samples as described in [66] (CD4+ T cells, CD8+ T cells, B cells, NK cells, monocytes and granulocytes) as a reference to estimate the relationship between cell types and methylation. After data pre-processing, we extracted ß-values of the 475 methylation sites available in our study that were overlapping with the 500 methylation sites showing the strongest relation with cell types in [66]. These data were used to estimate proportions of the six above-mentioned cell types in samples of the CHOP study using the R function projectWBC() provided by Andres Houseman, restricting the WBC estimates to positive numbers (option nonnegative = TRUE). The estimated WBC proportions were included as covariates in the regression models (see below).

### Statistical Analysis (EWAS)

Potential associations between methylation M-values at each CpG site and prenatal exposure to tobacco smoke beyond the 12^th^ week of gestation were determined by standard linear regression models with adjustment for sex, age at blood draw (months), study centre (Germany (DE), Italy (IT), Spain(ES), reference Belgium (BE)), maternal education (high = 12+ yrs. vs. low = basic schooling and middle (10 -<12 yrs. vs. low), postnatal smoking exposure at age 4 years (by mother (yes/no), by father(yes/no)), the estimated proportions of six major white blood cell types, CD4+ T cells, CD8+ T cells, B cells, NK cells, monocytes and granulocytes and the top 30 principal components (PC) derived from control probes on the HM450K platform. The latter were included to account for biological and technical noise and batch effects [65,66]. The former adjustment factors were selected based on previous studies [6–8] and to account for the study design of the CHOP study (multi-centre study with populations from 4 European countries) [9]. Model adjustment for child’s sex, age at blood draw, study centre, maternal education and postnatal smoke exposure was also conducted because lambda (λ) [67] was substantially improved from a model adjusted for cell mixture only (λ = 1.26), over a model adjusted for cell-mixture and 30 PC (λ = 0.98) to the final fully adjusted model (λ = 0.91).

An association of a differently methylated CpG site (M-values) with exposure was considered significant at the epigenome-wide level, if the false discovery rate (FDR) was below 0.05 [68]. EWAS-wide significance was also evaluated and illustrated by QQ-plots produced with module gcontrol2 of R-package gap. Volcano plots of the M-value based regression coefficients of the exposure variable were used to simultaneously illustrate effect size and EWAS significance.

### Functional characterization of methylated CpGs

Differentially methylated sites were annotated according to Illumina (www.illumina.com, HumanMethylation450_15017482_v1.csv) and viewed in the UCSC genome browser, GRCh37/h19 assembly [69]. The location of overlapping transcription factor binding sites, transcripts and chromatin modifications was assessed with ENCODE ChIP-seq from blood (GM12878, K562) and endothelial (HUVEC) cell lines [26]. Associations between transcription and DNA methylation at CpGs along the *MYO1G* locus was retrieved from the pan-cancer methylation database MethHC [25]. General and tobacco-related expression pattern of *CNTNAP2* and *FRMD4A* was assessed using Human Proteome Map [45] and Gene Expression Atlas at the European Bioinformatics Institute [42], respectively. All data were last retrieved in February 2016.

### Functional network analysis

Functional interpretation of our top five differentially methylated CpG sites (FDR<0.05) was performed using the GeneMANIA (http://genemania.org/) [11]. The network of 100 most strongly interacting genes with MYO1G, CNTNAP2 and FRMD4A was created based on physical interactions, co-expression, co-localization and shared protein domains The predicted interaction network was then analysed with the PANTHER (protein annotation through evolutionary relationship) classification system, version 10.0, released 2015-05-15, using the statistical overrepresentation test for gene ontology (GO) terms, protein classes and PANTHER pathways (http://pantherdb.org/index.jsp).

## Supporting Information

S1 Fig Chromatin and transcription along *MYO1G* locus in human hematopoietic (G12878, K562) and endothelial (HUVEC) cell lines (http://genome-euro.ucsc.edu/cgi-bin/hgTracks?db=hg19&lastVirtModeType=default&lastVirtModeExtraState=&virtModeType=default&virtMode=0&nonVirtPosition=&position=chr7%3A44998414-45023390&hgsid=212237736_XjE21xm20bpQVMUt2cQ6XC8wIHnm). In GM12878 cells, high (>60%) DNA methylation at the 3’ gene region associates with high transcription along the *MYO1G* locus, high promoter histone 3 lysine 27 (H3K27) acetylation and low (<20%) promoter DNA methylation. To the contrary, in K562 and HUVEC cells *MYO1G* locus is not transcribed and has a high (>60%) level of 3’ methylation (K562), no promoter H3K27 acetylation and high (>60%) promoter DNA methylation. Significantly methylated CpG sites in our study are among those in the red circle.
(TIF)

S2 Fig *MYO1G* transcription in bladderurothelial carcinoma (blca) inversely correlates with methylation of promoter/5’UTR CpGs (A) and directly correlates with methylation of CpGs at the 3’gene region (B) (http://methhc.mbc.nctu.edu.tw/php/correlation_probe.php?nm=NM_033054&tumor=blca&region=5UTR&levelmethod=mean&probe=cg21188037). Abbreviations: TSS200, 200 bp—long region upstream of the transcription start site (TSS); CGI, CpG island.
(TIFF)

S3 Fig Prenatal smoking-associated differentially methylated *MYO1G* 3’gene region overlaps with several transcription factor binding sites (http://genome-euro.ucsc.edu/cgi-bin/hgTracks?db=hg19&lastVirtModeType=default&lastVirtModeExtraState=&virtModeType=default&virtMode=0&nonVirtPosition=&position=chr7%3A45001441-45003907&hgsid=212237735_eHz7L4XBbhnr8oo1dAx556OaDHht). Significantly methylated CpG sites in our study are marked by red circles. Grey intensity of DNA binding factors is proportional to the maximum strength signal observed. Abbreviations: POLR2A, RNA polymerase II; MAX, Myc-associated factor; EZH2, enhancer of zeste homolog 2; TAF1, TBP-associated factor; USF2, upstream stimulatory factor 2; YY1, Yin and Yang 1 protein; RAD21, double-strand-break repair protein rad21 homolog; CTCF, Insulator protein (CCCTC-binding factor); USF1, upstream stimulatory factor 1.
(TIF)

S4 Fig Smoking-associated hypomethylated cg25949550 is located at the 5’ region of *CNTNAP2* and overlaps with binding sites of transcription repressors SIN3A, CTBP2, CTCF and REST (http://genome-euro.ucsc.edu/cgi-bin/hgTracks?hgS_doOtherUser=submit&hgS_otherUserName=suncanica&hgS_otherUserSessionName=Fig_S4_CNTNAP2). Significantly methylated CpG sites in our study are marked by the red circle. Abbreviations: SIN3A, SIN3 transcription regulator family member A; CTBP2, C-terminal-binding protein 2; CTCF, Insulator protein (CCCTC-binding factor); REST, RE1-silencing transcription factor.
(TIFF)

S5 Fig Prenatal smoking-hypermethylated cg11813497 is located at the *FRMD4A* promoter region next to the binding site for Gata-2 and a conserved binding site for the Tst1/Oct/Pou family of transcription factors (http://genome-euro.ucsc.edu/cgi-bin/hgTracks?hgS_doOtherUser=submit&hgS_otherUserName=suncanica&hgS_otherUserSessionName=Fig_S5_FRMD4A). Significantly methylated CpG sites in our study are marked by the red circle. *Abbreviations*: GATA2, Gata-binding protein 2; TST1; POU domain transcription factor 1, also known as Oct-6; FREAC7, forkhead box protein L1; FOXD3, forkhead box D3; HNF3B, hepatocyte nuclear factor 3-beta, also known as FOXA2; OCT1, octamer-binding protein 1, also known as POU2F1; MEF2, myocyte enhancer factor-2; GRE, glucocorticoid receptor; RSRFC4, myocyte enhancer factor-2a, known as MEF2A.
(TIFF)

S1 Table Comparison of prenatal smoking associated differentially methylated CpGs in children and adolescents. The table compares effect sizes, and uncorrected *P-*values across cohorts. Values that remained significant after applying Bonferroni (SEED, 0.05/26 = 1.92 x 10^−3^) or Benjamini-Hochberg (ALSPAC, CHOP and SYS) corrections are indicated in bold. *Abbreviations*: Chr, chromosome; Δβ, difference in DNA methylation level between offspring exposed and non-exposed to prenatal tobacco smoke; SEED, The Study to Explore Early Development; USA, the United States of America; CHOP, The European Childhood Obesity Project; BE, Belgium; DE, Germany; IT, Italy; ES, Spain; ALSPAC, The Avon Longitudinal study of Parents and Children; UK, the United Kingdom; SYS, The Saguenay Youth Study; Can, Canada. *Model adjustments*: SEED: Model adjusted for genetically estimated racial ancestry, autism spectrum disorder (ASD), sex, maternal education, maternal age, cell type proportions (Table 2, [8]). CHOP: Model adjusted for age + sex + maternal education + cell type proportions + 30 PC + study centre + postnatal parent smoking (Table 1, our study). ALSPAC-7.5 y.: Model adjusted for paternal smoking (Table S8, [7]). SYS: Model adjusted for age, sex, batch and blood cell fractions (Table 2 Model A, [6]). ALSPAC-17.7 y.: Model adjusted for paternal smoking (Table S9, [7]).
(XLSX)

S2 Table GeneMANIA gene list. (XLSX)

S3 Table PANTHER Pathways. (XLSX)

S4 Table GO biological process complete. (XLSX)

S5 Table GO molecular function complete. (XLSX)

S6 Table GO cellular component complete. (XLSX)

S7 Table PANTHER Protein Class. (XLSX)

## References

[1] Novakovic B, Ryan J, Pereira N, Boughton B, Craig JM, et al (2014) Postnatal stability, tissue, and time specific effects of AHRR methylation change in response to maternal smoking in pregnancy. Epigenetics 9: 377–386. 10.4161/epi.27248 24270552 PMC4053456

[2] Joubert BR, Haberg SE, Nilsen RM, Wang X, Vollset SE, et al (2012) 450K epigenome-wide scan identifies differential DNA methylation in newborns related to maternal smoking during pregnancy. Environ Health Perspect 120: 1425–1431. 10.1289/ehp.1205412 22851337 PMC3491949

[3] Markunas CA, Xu Z, Harlid S, Wade PA, Lie RT, et al (2014) Identification of DNA methylation changes in newborns related to maternal smoking during pregnancy. Environ Health Perspect 122: 1147–1153. 10.1289/ehp.1307892 24906187 PMC4181928

[4] Ivorra C, Fraga MF, Bayon GF, Fernandez AF, Garcia-Vicent C, et al (2015) DNA methylation patterns in newborns exposed to tobacco in utero. J Transl Med 13: 25 10.1186/s12967-015-0384-5 25623364 PMC4312439

[5] Kupers LK, Xu X, Jankipersadsing SA, Vaez A, la Bastide-van Gemert S, et al (2015) DNA methylation mediates the effect of maternal smoking during pregnancy on birthweight of the offspring. Int J Epidemiol. 10.1093/ije/dyv048 PMC4588868 25862628

[6] Lee KW, Richmond R, Hu P, French L, Shin J, et al (2015) Prenatal Exposure to Maternal Cigarette Smoking and DNA Methylation: Epigenome-Wide Association in a Discovery Sample of Adolescents and Replication in an Independent Cohort at Birth through 17 Years of Age. Environ Health Perspect 123: 193–199. 10.1289/ehp.1408614 25325234 PMC4314251

[7] Richmond RC, Simpkin AJ, Woodward G, Gaunt TR, Lyttleton O, et al (2015) Prenatal exposure to maternal smoking and offspring DNA methylation across the lifecourse: findings from the Avon Longitudinal Study of Parents and Children (ALSPAC). Hum Mol Genet: 17. 10.1093/hmg/ddu739 PMC4380069 25552657

[8] Ladd-Acosta C, Shu C, Lee BK, Gidaya N, Singer A, et al (2016) Presence of an epigenetic signature of prenatal cigarette smoke exposure in childhood. Environ Res 144: 139–148. 10.1016/j.envres.2015.11.014 26610292 PMC4915563

[9] Koletzko B, von Kries R, Closa R, Escribano J, Scaglioni S, et al (2009) Lower protein in infant formula is associated with lower weight up to age 2 y: a randomized clinical trial. Am J Clin Nutr 89: 1836–1845. 10.3945/ajcn.2008.27091 19386747

[10] Mi H, Dong Q, Muruganujan A, Gaudet P, Lewis S, et al (2010) PANTHER version 7: improved phylogenetic trees, orthologs and collaboration with the Gene Ontology Consortium. Nucleic Acids Res 38: D204–210. 10.1093/nar/gkp1019 20015972 PMC2808919

[11] Warde-Farley D, Donaldson SL, Comes O, Zuberi K, Badrawi R, et al (2010) The GeneMANIA prediction server: biological network integration for gene prioritization and predicting gene function. Nucleic Acids Res 38: W214–220. 10.1093/nar/gkq537 20576703 PMC2896186

[12] Breton CV, Siegmund KD, Joubert BR, Wang X, Qui W, et al (2014) Prenatal tobacco smoke exposure is associated with childhood DNA CpG methylation. PLoS One 9: e99716 10.1371/journal.pone.0099716 24964093 PMC4070909

[13] Key AP, Ferguson M, Molfese DL, Peach K, Lehman C, et al (2007) Smoking during pregnancy affects speech-processing ability in newborn infants. Environ Health Perspect 115: 623–629. 17450234 10.1289/ehp.9521 PMC1852679

[14] Motlagh MG, Sukhodolsky DG, Landeros-Weisenberger A, Katsovich L, Thompson N, et al (2011) Adverse effects of heavy prenatal maternal smoking on attentional control in children with ADHD. J Atten Disord 15: 593–603. 10.1177/1087054710374576 20616372 PMC3974616

[15] Kalkbrenner AE, Braun JM, Durkin MS, Maenner MJ, Cunniff C, et al (2012) Maternal smoking during pregnancy and the prevalence of autism spectrum disorders, using data from the autism and developmental disabilities monitoring network. Environ Health Perspect 120: 1042–1048. 10.1289/ehp.1104556 22534110 PMC3404663

[16] Herberth G, Bauer M, Gasch M, Hinz D, Roder S, et al (2014) Maternal and cord blood miR-223 expression associates with prenatal tobacco smoke exposure and low regulatory T-cell numbers. J Allergy Clin Immunol 133: 543–550. 10.1016/j.jaci.2013.06.036 23978443

[17] Akuete K, Oh SS, Thyne S, Rodriguez-Santana JR, Chapela R, et al (2011) Ethnic variability in persistent asthma after in utero tobacco exposure. Pediatrics 128: e623–630. 10.1542/peds.2011-0640 21859918 PMC3164096

[18] Behl M, Rao D, Aagaard K, Davidson TL, Levin ED, et al (2013) Evaluation of the association between maternal smoking, childhood obesity, and metabolic disorders: a national toxicology program workshop review. Environ Health Perspect 121: 170–180. 10.1289/ehp.1205404 23232494 PMC3569686

[19] O'Callaghan FV, Al Mamun A, O'Callaghan M, Alati R, Najman JM, et al (2009) Maternal smoking during pregnancy predicts nicotine disorder (dependence or withdrawal) in young adults—a birth cohort study. Aust N Z J Public Health 33: 371–377. 10.1111/j.1753-6405.2009.00410.x 19689599

[20] Besingi W, Johansson A (2014) Smoke-related DNA methylation changes in the etiology of human disease. Hum Mol Genet 23: 2290–2297. 10.1093/hmg/ddt621 24334605

[21] Elliott HR, Tillin T, McArdle WL, Ho K, Duggirala A, et al (2014) Differences in smoking associated DNA methylation patterns in South Asians and Europeans. Clin Epigenetics 6: 4 10.1186/1868-7083-6-4 24485148 PMC3915234

[22] Guida F, Sandanger TM, Castagne R, Campanella G, Polidoro S, et al (2015) Dynamics of smoking-induced genome-wide methylation changes with time since smoking cessation. Hum Mol Genet 24: 2349–2359. 10.1093/hmg/ddu751 25556184 PMC4380075

[23] Zeilinger S, Kuhnel B, Klopp N, Baurecht H, Kleinschmidt A, et al (2013) Tobacco smoking leads to extensive genome-wide changes in DNA methylation. PLoS One 8: e63812 10.1371/journal.pone.0063812 23691101 PMC3656907

[24] Zhu X, Li J, Deng S, Yu K, Liu X, et al (2016) Genome-Wide Analysis of DNA Methylation and Cigarette Smoking in Chinese. Environ Health Perspect. 10.1289/ehp.1509834 PMC4937856 26756918

[25] Huang WY, Hsu SD, Huang HY, Sun YM, Chou CH, et al (2015) MethHC: a database of DNA methylation and gene expression in human cancer. Nucleic Acids Res 43: D856–861. 10.1093/nar/gku1151 25398901 PMC4383953

[26] Consortium EP (2012) An integrated encyclopedia of DNA elements in the human genome. Nature 489: 57–74. 10.1038/nature11247 22955616 PMC3439153

[27] Roadmap Epigenomics C, Kundaje A, Meuleman W, Ernst J, Bilenky M, et al (2015) Integrative analysis of 111 reference human epigenomes. Nature 518: 317–330. 10.1038/nature14248 25693563 PMC4530010

[28] Pierce RA, Field ED, Mutis T, Golovina TN, Von Kap-Herr C, et al (2001) The HA-2 minor histocompatibility antigen is derived from a diallelic gene encoding a novel human class I myosin protein. J Immunol 167: 3223–3230. 11544309 10.4049/jimmunol.167.6.3223

[29] Jones PA (2012) Functions of DNA methylation: islands, start sites, gene bodies and beyond. Nat Rev Genet 13: 484–492. 10.1038/nrg3230 22641018

[30] Vire E, Brenner C, Deplus R, Blanchon L, Fraga M, et al (2006) The Polycomb group protein EZH2 directly controls DNA methylation. Nature 439: 871–874. 16357870 10.1038/nature04431

[31] Kornblihtt AR (2012) CTCF: from insulators to alternative splicing regulation. Cell Res 22: 450–452. 10.1038/cr.2012.22 22310241 PMC3292291

[32] Naik P, Cucullo L (2015) Pathobiology of tobacco smoking and neurovascular disorders: untied strings and alternative products. Fluids Barriers CNS 12: 25 10.1186/s12987-015-0022-x 26520792 PMC4628383

[33] Bakker H, Jaddoe VW (2011) Cardiovascular and metabolic influences of fetal smoke exposure. Eur J Epidemiol 26: 763–770. 10.1007/s10654-011-9621-2 21994150 PMC3218270

[34] Heng TS, Painter MW, Immunological Genome Project C (2008) The Immunological Genome Project: networks of gene expression in immune cells. Nat Immunol 9: 1091–1094. 10.1038/ni1008-1091 18800157

[35] Gerard A, Patino-Lopez G, Beemiller P, Nambiar R, Ben-Aissa K, et al (2014) Detection of rare antigen-presenting cells through T cell-intrinsic meandering motility, mediated by Myo1g. Cell 158: 492–505. 10.1016/j.cell.2014.05.044 25083865 PMC4119593

[36] Maravillas-Montero JL, Lopez-Ortega O, Patino-Lopez G, Santos-Argumedo L (2014) Myosin 1g regulates cytoskeleton plasticity, cell migration, exocytosis, and endocytosis in B lymphocytes. Eur J Immunol 44: 877–886. 10.1002/eji.201343873 24310084

[37] Olety B, Walte M, Honnert U, Schillers H, Bahler M (2010) Myosin 1G (Myo1G) is a haematopoietic specific myosin that localises to the plasma membrane and regulates cell elasticity. FEBS Lett 584: 493–499. 10.1016/j.febslet.2009.11.096 19968988

[38] Kable JA, Coles CD, Lynch ME, Carroll J (2009) The impact of maternal smoking on fast auditory brainstem responses. Neurotoxicol Teratol 31: 216–224. 10.1016/j.ntt.2009.02.002 19224709 PMC2693298

[39] Dumont RA, Zhao YD, Holt JR, Bahler M, Gillespie PG (2002) Myosin-I isozymes in neonatal rodent auditory and vestibular epithelia. J Assoc Res Otolaryngol 3: 375–389. 12486594 10.1007/s101620020049 PMC3202438

[40] Zadro C, Alemanno MS, Bellacchio E, Ficarella R, Donaudy F, et al (2009) Are MYO1C and MYO1F associated with hearing loss? Biochim Biophys Acta 1792: 27–32. 10.1016/j.bbadis.2008.10.017 19027848

[41] Wan ES, Qiu W, Baccarelli A, Carey VJ, Bacherman H, et al (2012) Cigarette smoking behaviors and time since quitting are associated with differential DNA methylation across the human genome. Hum Mol Genet 21: 3073–3082. 10.1093/hmg/dds135 22492999 PMC3373248

[42] Kapushesky M, Emam I, Holloway E, Kurnosov P, Zorin A, et al (2010) Gene expression atlas at the European bioinformatics institute. Nucleic Acids Res 38: D690–698. 10.1093/nar/gkp936 19906730 PMC2808905

[43] Hussain M, Rao M, Humphries AE, Hong JA, Liu F, et al (2009) Tobacco smoke induces polycomb-mediated repression of Dickkopf-1 in lung cancer cells. Cancer Res 69: 3570–3578. 10.1158/0008-5472.CAN-08-2807 19351856 PMC8374472

[44] Jorgensen E, Stinson A, Shan L, Yang J, Gietl D, et al (2008) Cigarette smoke induces endoplasmic reticulum stress and the unfolded protein response in normal and malignant human lung cells. BMC Cancer 8: 229 10.1186/1471-2407-8-229 18694499 PMC2527015

[45] Kim MS, Pinto SM, Getnet D, Nirujogi RS, Manda SS, et al (2014) A draft map of the human proteome. Nature 509: 575–581. 10.1038/nature13302 24870542 PMC4403737

[46] Krumbiegel M, Pasutto F, Schlotzer-Schrehardt U, Uebe S, Zenkel M, et al (2011) Genome-wide association study with DNA pooling identifies variants at CNTNAP2 associated with pseudoexfoliation syndrome. Eur J Hum Genet 19: 186–193. 10.1038/ejhg.2010.144 20808326 PMC3025781

[47] Rodenas-Cuadrado P, Ho J, Vernes SC (2014) Shining a light on CNTNAP2: complex functions to complex disorders. Eur J Hum Genet 22: 171–178. 10.1038/ejhg.2013.100 23714751 PMC3895625

[48] Bock C, Beerman I, Lien WH, Smith ZD, Gu H, et al (2012) DNA methylation dynamics during in vivo differentiation of blood and skin stem cells. Mol Cell 47: 633–647. 10.1016/j.molcel.2012.06.019 22841485 PMC3428428

[49] Veljkovic E, Jiricny J, Menigatti M, Rehrauer H, Han W (2011) Chronic exposure to cigarette smoke condensate in vitro induces epithelial to mesenchymal transition-like changes in human bronchial epithelial cells, BEAS-2B. Toxicol In Vitro 25: 446–453. 10.1016/j.tiv.2010.11.011 21095227

[50] Cao J, Dwyer JB, Mangold JE, Wang J, Wei J, et al (2011) Modulation of cell adhesion systems by prenatal nicotine exposure in limbic brain regions of adolescent female rats. Int J Neuropsychopharmacol 14: 157–174. 10.1017/S1461145710000179 20196919 PMC5575906

[51] Ikenouchi J, Umeda M (2010) FRMD4A regulates epithelial polarity by connecting Arf6 activation with the PAR complex. Proc Natl Acad Sci U S A 107: 748–753. 10.1073/pnas.0908423107 20080746 PMC2818900

[52] Goldie SJ, Mulder KW, Tan DW, Lyons SK, Sims AH, et al (2012) FRMD4A upregulation in human squamous cell carcinoma promotes tumor growth and metastasis and is associated with poor prognosis. Cancer Res 72: 3424–3436. 10.1158/0008-5472.CAN-12-0423 22564525 PMC3428932

[53] Denisenko-Nehrbass N, Oguievetskaia K, Goutebroze L, Galvez T, Yamakawa H, et al (2003) Protein 4.1B associates with both Caspr/paranodin and Caspr2 at paranodes and juxtaparanodes of myelinated fibres. Eur J Neurosci 17: 411–416. 12542678 10.1046/j.1460-9568.2003.02441.x

[54] Yoon D, Kim YJ, Cui WY, Van der Vaart A, Cho YS, et al (2012) Large-scale genome-wide association study of Asian population reveals genetic factors in FRMD4A and other loci influencing smoking initiation and nicotine dependence. Hum Genet 131: 1009–1021. 10.1007/s00439-011-1102-x 22006218 PMC4275569

[55] Abreu-Villaca Y, Seidler FJ, Tate CA, Cousins MM, Slotkin TA (2004) Prenatal nicotine exposure alters the response to nicotine administration in adolescence: effects on cholinergic systems during exposure and withdrawal. Neuropsychopharmacology 29: 879–890. 14970833 10.1038/sj.npp.1300401

[56] Weber M, Grote V, Closa-Monasterolo R, Escribano J, Langhendries JP, et al (2014) Lower protein content in infant formula reduces BMI and obesity risk at school age: follow-up of a randomized trial. Am J Clin Nutr. 10.3945/ajcn.113.064071 24622805

[57] Richmond RC, Simpkin AJ, Woodward G, Gaunt TR, Lyttleton O, et al (2015) Prenatal exposure to maternal smoking and offspring DNA methylation across the lifecourse: findings from the Avon Longitudinal Study of Parents and Children (ALSPAC). Hum Mol Genet 24: 2201–2217. 10.1093/hmg/ddu739 25552657 PMC4380069

[58] Bibikova M, Barnes B, Tsan C, Ho V, Klotzle B, et al (2011) High density DNA methylation array with single CpG site resolution. Genomics 98: 288–295. 10.1016/j.ygeno.2011.07.007 21839163

[59] Du P, Zhang X, Huang CC, Jafari N, Kibbe WA, et al (2010) Comparison of Beta-value and M-value methods for quantifying methylation levels by microarray analysis. BMC Bioinformatics 11: 587 10.1186/1471-2105-11-587 21118553 PMC3012676

[60] Touleimat N, Tost J (2012) Complete pipeline for Infinium((R)) Human Methylation 450K BeadChip data processing using subset quantile normalization for accurate DNA methylation estimation. Epigenomics 4: 325–341. 10.2217/epi.12.21 22690668

[61] Teschendorff AE, Marabita F, Lechner M, Bartlett T, Tegner J, et al (2013) A beta-mixture quantile normalization method for correcting probe design bias in Illumina Infinium 450 k DNA methylation data. Bioinformatics 29: 189–196. 10.1093/bioinformatics/bts680 23175756 PMC3546795

[62] Marabita F, Almgren M, Lindholm ME, Ruhrmann S, Fagerstrom-Billai F, et al (2013) An evaluation of analysis pipelines for DNA methylation profiling using the Illumina HumanMethylation450 BeadChip platform. Epigenetics 8: 333–346. 10.4161/epi.24008 23422812 PMC3669124

[63] Wahl S, Fenske N, Zeilinger S, Suhre K, Gieger C, et al (2014) On the potential of models for location and scale for genome-wide DNA methylation data. BMC Bioinformatics 15: 232 10.1186/1471-2105-15-232 24994026 PMC4227139

[64] Chen YA, Lemire M, Choufani S, Butcher DT, Grafodatskaya D, et al (2013) Discovery of cross-reactive probes and polymorphic CpGs in the Illumina Infinium HumanMethylation450 microarray. Epigenetics 8: 203–209. 10.4161/epi.23470 23314698 PMC3592906

[65] Lehne B, Drong AW, Loh M, Zhang W, Scott WR, et al (2015) A coherent approach for analysis of the Illumina HumanMethylation450 BeadChip improves data quality and performance in epigenome-wide association studies. Genome Biol 16: 37 10.1186/s13059-015-0600-x 25853392 PMC4365767

[66] Houseman EA, Accomando WP, Koestler DC, Christensen BC, Marsit CJ, et al (2012) DNA methylation arrays as surrogate measures of cell mixture distribution. BMC Bioinformatics 13: 86 10.1186/1471-2105-13-86 22568884 PMC3532182

[67] Devlin B, Roeder K (1999) Genomic Control for Association Studies. Biometrics 55: 997–1004. 11315092 10.1111/j.0006-341x.1999.00997.x

[68] Benjamini Y, Hochberg Y (1995) Controlling the False Discovery Rate—a Practical and Powerful Approach to Multiple Testing. Journal of the Royal Statistical Society Series B-Methodological 57: 289–300.

[69] Kent WJ, Sugnet CW, Furey TS, Roskin KM, Pringle TH, et al (2002) The human genome browser at UCSC. Genome Res 12: 996–1006. 12045153 10.1101/gr.229102 PMC186604

