# Supplementary figures and images for: Maternal Smoking during Pregnancy and DNA-Methylation in Children at Age 5.5 Years: Epigenome-Wide-Analysis in the European Childhood Obesity Project (CHOP)-Study
Source: PLoS One. 2016 May 12;11(5):e0155554. doi: 10.1371/journal.pone.0155554 (PMC4865176; doi:10.1371/journal.pone.0155554)

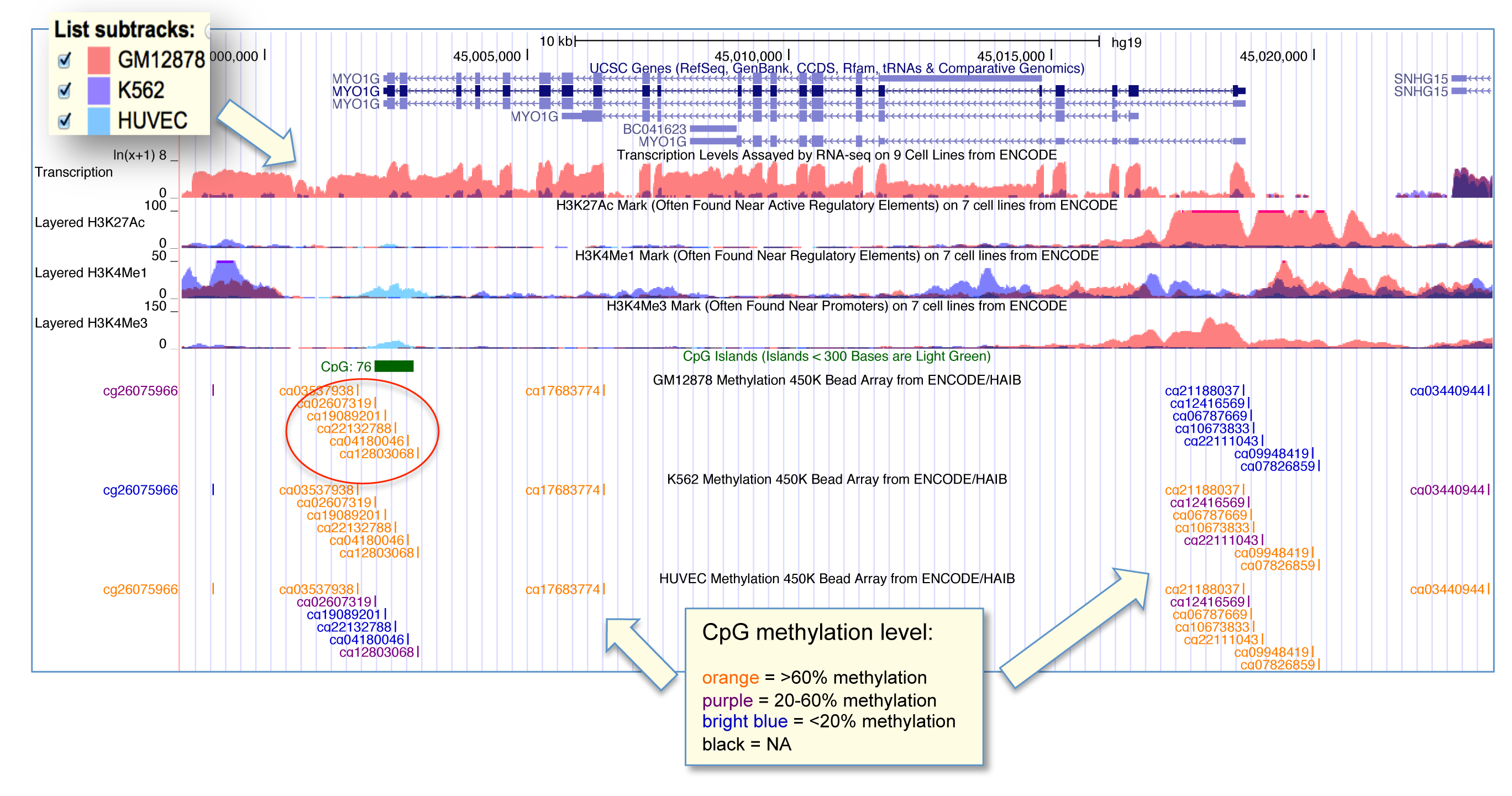

Supplement: S1 Fig — In GM12878 cells, high (>60%) DNA methylation at the 3’ gene region associates with high transcription along the MYO1G locus, high promoter histone 3 lysine 27 (H3K27) acetylation and low (<20%) promoter DNA methylation. To the contrary, in K562 and HUVEC cells MYO1G locus is not transcribed and has a high (>60%) level of 3’ methylation (K562), no promoter H3K27 acetylation and high (>60%) promoter DNA methylation. Significantly methylated CpG sites in our study are among those in the red circle. (TIF) [file pone.0155554.s001.tif]

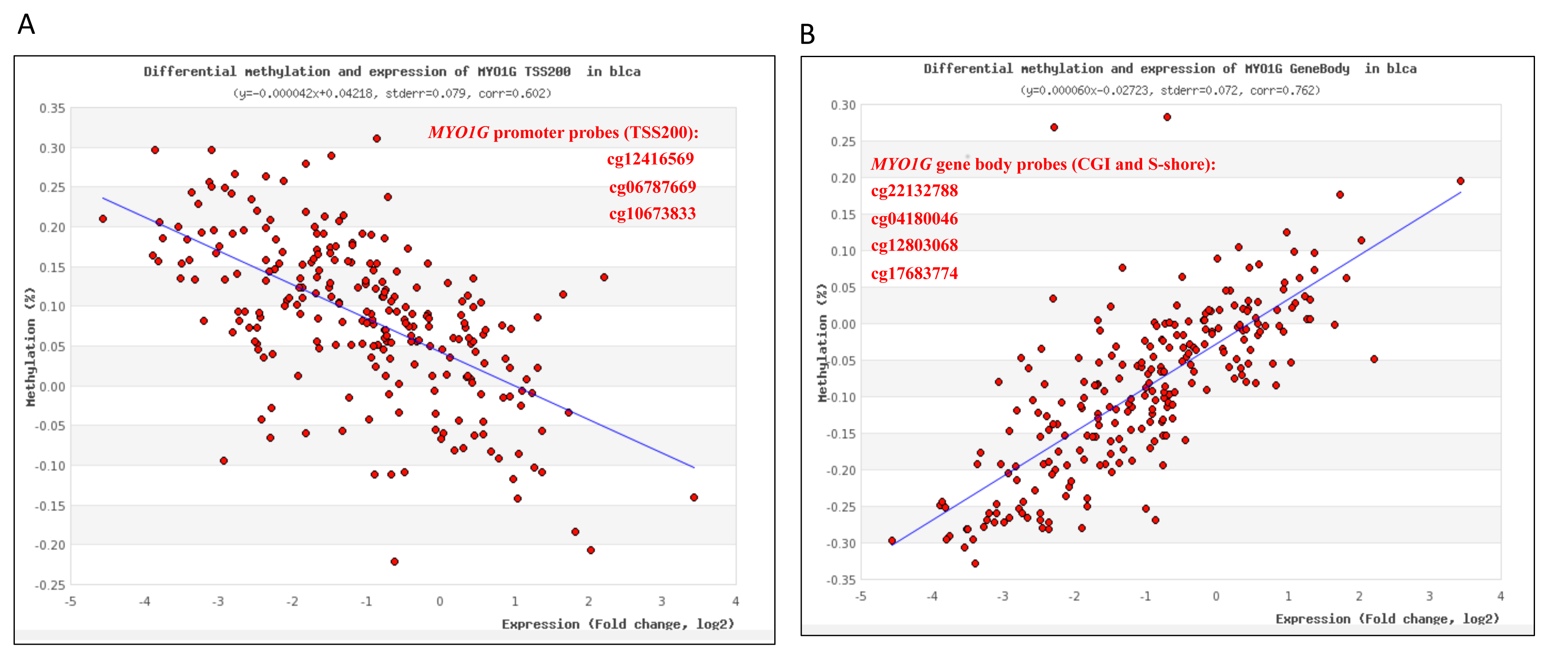

Supplement: S2 Fig — MYO1G transcription in bladderurothelial carcinoma (blca) inversely correlates with methylation of promoter/5’UTR CpGs (A) and directly correlates with methylation of CpGs at the 3’gene region (B) (http://methhc.mbc.nctu.edu.tw/php/correlation_probe.php?nm=NM_033054&tumor=blca®ion=5UTR&levelmethod=mean&probe=cg21188037). Abbreviations: TSS200, 200 bp—long region upstream of the transcription start site (TSS); CGI, CpG island. (TIFF) [file pone.0155554.s002.tiff]

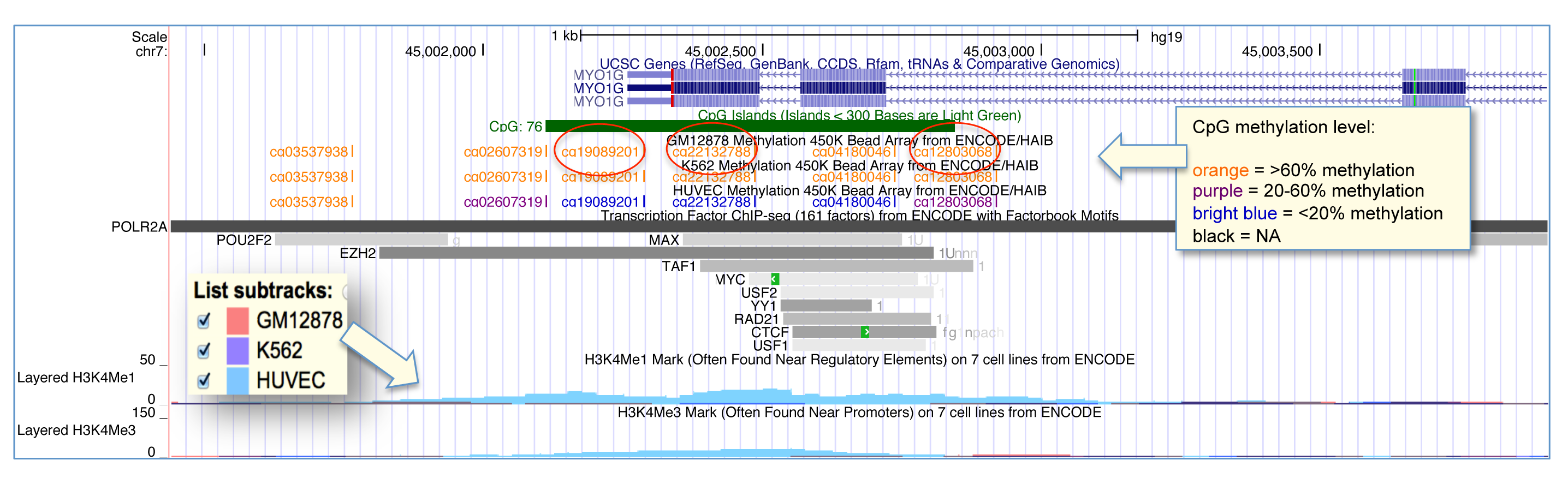

Supplement: S3 Fig — Significantly methylated CpG sites in our study are marked by red circles. Grey intensity of DNA binding factors is proportional to the maximum strength signal observed. Abbreviations: POLR2A, RNA polymerase II; MAX, Myc-associated factor; EZH2, enhancer of zeste homolog 2; TAF1, TBP-associated factor; USF2, upstream stimulatory factor 2; YY1, Yin and Yang 1 protein; RAD21, double-strand-break repair protein rad21 homolog; CTCF, Insulator protein (CCCTC-binding factor); USF1, upstream stimulatory factor 1. (TIF) [file pone.0155554.s003.tif]

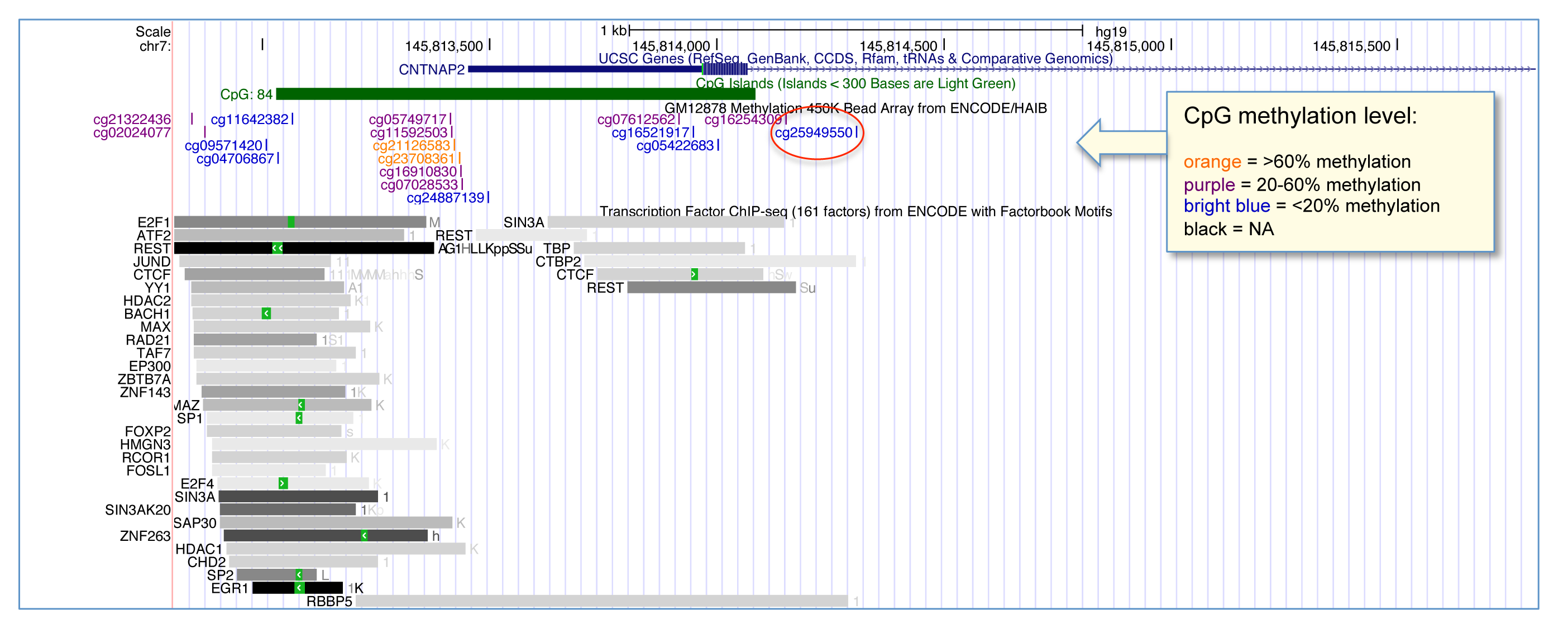

Supplement: S4 Fig — Significantly methylated CpG sites in our study are marked by the red circle. Abbreviations: SIN3A, SIN3 transcription regulator family member A; CTBP2, C-terminal-binding protein 2; CTCF, Insulator protein (CCCTC-binding factor); REST, RE1-silencing transcription factor. (TIFF) [file pone.0155554.s004.tiff]

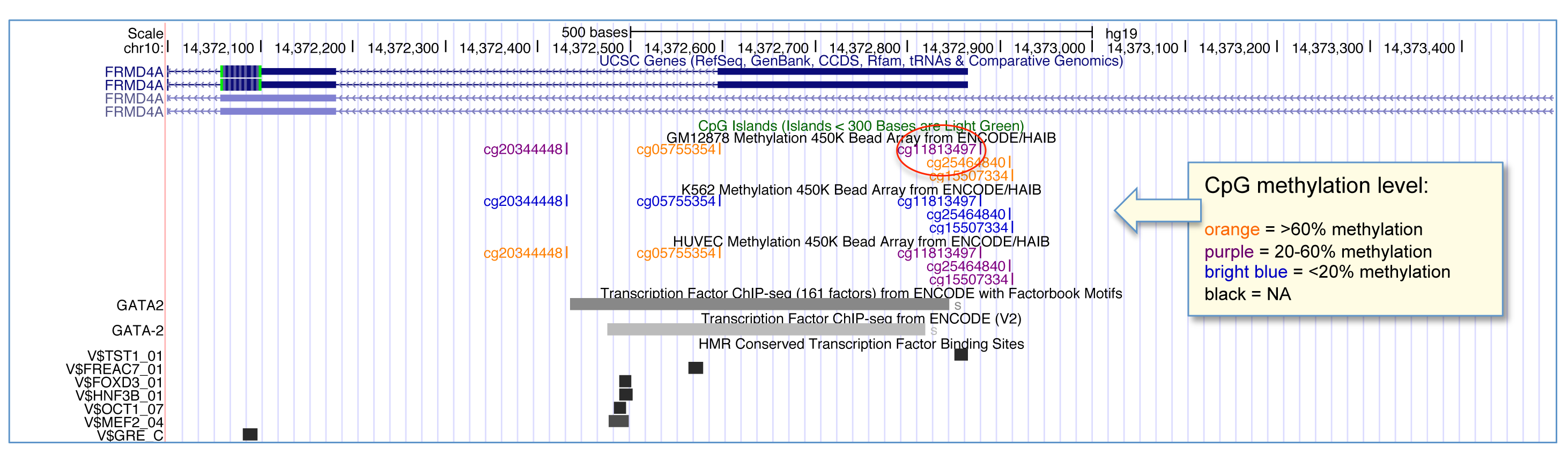

Supplement: S5 Fig — Significantly methylated CpG sites in our study are marked by the red circle. Abbreviations: GATA2, Gata-binding protein 2; TST1; POU domain transcription factor 1, also known as Oct-6; FREAC7, forkhead box protein L1; FOXD3, forkhead box D3; HNF3B, hepatocyte nuclear factor 3-beta, also known as FOXA2; OCT1, octamer-binding protein 1, also known as POU2F1; MEF2, myocyte enhancer factor-2; GRE, glucocorticoid receptor; RSRFC4, myocyte enhancer factor-2a, known as MEF2A. (TIFF) [file pone.0155554.s005.tiff]
